# Effect of Media on Facial Plastic Surgery in Saudi Arabia

**DOI:** 10.7759/cureus.6232

**Published:** 2019-11-25

**Authors:** Badi F Aldosari, Mohmmed Alkarzae, Reham Almuhaya, Razan Aldhahri, Hana Alrashid

**Affiliations:** 1 Otolaryngology, King Saud University, Riyadh, SAU; 2 Otolaryngology, Security Forces Hospital, Riyadh, SAU; 3 Otolaryngology - Head and Neck Surgery, Princess Nourah Bint Abdulrahman University, Riyadh, SAU; 4 Otolaryngology - Head and Neck Surgery, King Saud University, Riyadh, SAU

**Keywords:** cosmetic surgery, social media, advertisement

## Abstract

Objectives

To evaluate the effect of social media, TV shows, plastic surgeons' self-advertisement, and before-and-after cosmetic surgery photos of patients who actually visited the clinic to seek a consultation or intervention.

Methods

This is a cross-sectional study; institutional review board approval was granted in 2018. This study was conducted among patients attending cosmetic clinics at King Abdulaziz University Hospital in Riyadh, Saudi Arabia. The questionnaire is composed of socio-demographic data and about the reason for the trending of plastic surgeries.

Results

Three hundred and ninety-nine patients participated in the study. Of all participants, 60.4% agreed on the impact of the surgeon’s self-advertisement in the trending of plastic surgeries; 53.4% said yes to cosmetic television programs having an effect on the trend of plastic surgeries; 65.7% of the participants answered yes to before-and-after pictures of social media having an effect on the trend of cosmetic procedures; and 54.1% of the participants answered yes to wanting to look better in selfies as a reason for the rise of cosmetic surgery.

Conclusion

The results of this study have shown that the majority of patients visiting plastic surgery clinics were positively affected, but not exclusively, by media coverage of cosmetic surgery results.

## Introduction

Cosmetic surgery, by definition, focuses on enhancing the appearance, including surgical procedures, such as blepharoplasty, rhinoplasty, and breast augmentation, as well as nonsurgical procedures such as chemical peels and botulinum toxin injections. Plastic surgery is, by definition, dealing mainly with the surgical repair or restoration of an injured, lost, diseased, defective, or misshapen part or area, and it typically involves tissue grafting (such as skin or cartilage) from one part of the body to another [1]. The total number of plastic surgeries is constantly increasing over the years. According to the American Society of Plastic Surgery (ASPS), nearly 17.5 million cosmetic procedures were performed in 2017, with rhinoplasty and blepharoplasty being among the top five procedures performed in 2016-2017 [2]. The rate of increase in cosmetic procedures from 2012 to 2017 for both men and women was estimated to reach 65.2% [3]. Moreover, the emergence of new products and technology evolvement seems to boost steady growth in US cosmetic surgery rates [2]. According to a study conducted by the International Society of Aesthetic Plastic Surgery (ISAPS), Saudi Arabia ranks 29th among the top 30 countries, with the highest rates of cosmetic procedures performed worldwide [4]. Generally, it was found that higher incomes, lower procedure costs, media coverage, and positive attitude towards cosmetic procedures have positively affected the rise of procedures performed annually [5].

Social media is considered one of the newer aspects of the Internet. It is a common term for web-based applications that involve interactive communication through web-based technologies. This new and unique connection tool offers a way to disseminate information to a specific audience in real time. Popular examples include Internet forums, weblogs, and social network sites such as Facebook and Twitter [6-7]. Besides social media, the surge in media coverage through TV reality shows, which are programs that film people doing unscripted activities to entertain the public, has changed the perception of ideal beauty standards [8-9]. Slevec and Tiggemann previously reported that the middle-aged female audience of such programs that are centered upon appearances have been associated with higher levels of body dissatisfaction [10]. Moreover, the public has been influenced by celebrities in terms of copying their hairstyles, fashion trends, and even body types [11]. Thus, researchers have found an increase in media fascination with celebrities’ elective procedures reporting [12]. Accordingly, researchers found that celebrity worship of an individual whose body was admired predicted the likelihood of cosmetic surgery [13]. The enhancement in media coverage and the spread of TV reality shows have been widely believed to impact people’s decision to undergo surgery. In this regard, Crockett, Pruzinsky, and Persing surveyed people considering cosmetic surgery about their makeover TV shows viewership and 79% of them claimed that watching such shows has influenced them to seek cosmetic surgery [14]. Along with that, there has been a noticeable boost in cosmetic surgery advertisements [15]. Therefore, research showed that the vast majority of women have been exposed to cosmetic surgery advertisements, and they suggested that the rise in cosmetic surgery advertisements has negatively impacted women's appearance-related security and body satisfaction [16-17].

As the demand for elective cosmetic surgeries continues to rise and little research has examined the extent to which cosmetic surgery advertisement has influenced individuals’ desire for self-change; it is important to study the effect of the media and how it motivates the public to undergo cosmetic procedures. In addition, since there has been a lack of knowledge locally about whether media and advertisements affect the public or not, this created a persistent need to establish a baseline about their effect to fully understand the overall picture. Therefore, this study aims to evaluate the effect of social media, TV shows, plastic surgeons’ self-advertisement, and before-and-after cosmetic surgery photos on the patients who actually visited the clinic to seek a consultation or intervention.

## Materials and methods

Study design

It is a quantitative, observational, cross-sectional study using an anonymous paper and electronic-based questionnaire.

Participants

The participants of this study were 399 Saudi patients who attended the cosmetic clinic from March 2019 to August 2019 in order to undergo various cosmetic procedures and interventions.

Demographics

All participants provided their demographic details, including age, gender, educational level, marital status, financial income, and monthly family income.

Measures

The questionnaire consisted of two parts. The first part was about the socio-demographic data, which was made up of 10 close-ended questions (age, gender, education status, marital status, financial income, monthly income, undergoing cosmetic interventions and plastic surgeries before, wish/plan to do plastic interventions, and numbers of selfies taken per day). The second part was focused on close-ended questions about the reason behind the trending of plastic surgeries.

Statistical Package for Social Sciences (SPSS) version 23.0 (IBM Corp., Armonk, NY) was used for Statistical analysis. A p-value of less than 0.05 was considered statistically significant. The chi-square test was used to determine the statistical difference between categorical groups or proportions.

## Results

Plastic surgeons’ self-advertisement

The participants were asked if plastic surgeons’ self-advertisement of their work had an effect on the trending of plastic surgeries. Overall, a total of 60.4% of the participants answered "yes." According to age, 6.3% of them were less than 20 years of age; 19.5% were aged between 21 and 30 years; 14.5% were aged between 31 and 40 years; 15.8% were aged between 41 and 50 years; 4.3% of them were aged more than 51 years of age, with a significant p-value of 0.013 (Table 1). Based on gender, 44.1% of males and 63.2% of females answered with "yes."

**Table 1:** The participants’ response (age-stratified) towards the impact of surgeons’ self-advertisement on the overall trending of plastic surgeries

Age	Less than 20	Count	25	15	40
		% within Age	62.5%	37.5%	100.0%
		% of Total	6.3%	3.8%	10.0%
	21-30	Count	78	66	144
		% within Age	54.2%	45.8%	100.0%
		% of Total	19.5%	16.5%	36.1%
	31-40	Count	58	43	101
		% within Age	57.4%	42.6%	100.0%
		% of Total	14.5%	10.8%	25.3%
	41-50	Count	63	19	82
		% within Age	76.8%	23.2%	100.0%
		% of Total	15.8%	4.8%	20.6%
	more than 51	Count	17	15	32
		% within Age	53.1%	46.9%	100.0%
		% of Total	4.3%	3.8%	8.0%
Total		Count	241	158	399
		% within Age	60.4%	39.6%	100.0%
		% of Total	60.4%	39.6%	100.0%

Cosmetic television programs

The participants were asked if watching cosmetic television programs had an effect on the trending of plastic surgeries. Overall, more than half of the surveyed population (53.4%) answered "yes." According to age, 4% of them were aged less than 20 years of age; 15.3% were aged between 21 and 30 years; 16.3% were aged between 31 and 40 years; 13% were aged between 41 and 50 years: 4.8% were older than 51 years of age, with a significant p-value of 0.001 (Table 2).

**Table 2:** The participants’ response (age-stratified) towards the impact of cosmetic television programs on the overall trending of plastic surgeries

Age	Less than 20	Count	16	24	40
		% within Age	40.0%	60.0%	100.0%
		% of Total	4.0%	6.0%	10.0%
	21-30	Count	61	83	144
		% within Age	42.4%	57.6%	100.0%
		% of Total	15.3%	20.8%	36.1%
	31-40	Count	65	36	101
		% within Age	64.4%	35.6%	100.0%
		% of Total	16.3%	9.0%	25.3%
	41-50	Count	52	30	82
		% within Age	63.4%	36.6%	100.0%
		% of Total	13.0%	7.5%	20.6%
	more than 51	Count	19	13	32
		% within Age	59.4%	40.6%	100.0%
		% of Total	4.8%	3.3%	8.0%
Total		Count	213	186	399
		% within Age	53.4%	46.6%	100.0%
		% of Total	53.4%	46.6%	100.0%

Before-and-after pictures on social media

The study participants were asked if before-and-after photos on social media had affected their decision to undergo plastic surgery or not. Overall, a total of 65.7% of the participants answered "yes." According to age, 6.5% of the participants were less than 20 years of age, 21.8% were aged between 21 to 30 years, 17.8% were aged between 31 and 40 years, 15.5% were aged between 41 and 50 years; 4% were older than 51 years of age, with a significant p-value of 0.044 (Table 3). Based on gender, a total of 69.7% of the females and 42.4% of the males answered "yes."

**Table 3:** The participants’ response (age-stratified) towards the impact of pre- and post-surgical photos on social media on the overall trending of plastic surgeries

Age	Less than 20	Count	26	14	40
		% within Age	65.0%	35.0%	100.0%
		% of Total	6.5%	3.5%	10.0%
	21-30	Count	87	57	144
		% within Age	60.4%	39.6%	100.0%
		% of Total	21.8%	14.3%	36.1%
	31-40	Count	71	30	101
		% within Age	70.3%	29.7%	100.0%
		% of Total	17.8%	7.5%	25.3%
	41-50	Count	62	20	82
		% within Age	75.6%	24.4%	100.0%
		% of Total	15.5%	5.0%	20.6%
	more than 51	Count	16	16	32
		% within Age	50.0%	50.0%	100.0%
		% of Total	4.0%	4.0%	8.0%
Total		Count	262	137	399
		% within Age	65.7%	34.3%	100.0%
		% of Total	65.7%	34.3%	100.0%

To look better in selfies and pictures

The participants were asked if wanting to look better in selfies, and pictures had an effect on the trending of plastic surgeries. A total of 54.1% of the participants answered "yes," and this finding had a significant p-value of 0.003. Based on their educational level, 37.1% of them were bachelor’s students, 12.3% of them were high-school students, 3% of them were master's degree students, and 1.8% of them were Ph.D. students. According to age, 8% of them were less than 20 years of age, 17.8% were aged between 21 to 30 years, 13% were aged between 31 to 40 years, 11.3% were aged between 41 to 50 years, and 4% were older than 51 years of age, with a significant p-value of 0.013 (Table 4).

**Table 4:** The participants’ response (age-stratified) towards the impact of surgeons’ self-advertisement on the overall trending of plastic surgeries

Age	Less than 20	Count	32	8	40
		% within Age	80.0%	20.0%	100.0%
		% of Total	8.0%	2.0%	10.0%
	21-30	Count	71	73	144
		% within Age	49.3%	50.7%	100.0%
		% of Total	17.8%	18.3%	36.1%
	31-40	Count	52	49	101
		% within Age	51.5%	48.5%	100.0%
		% of Total	13.0%	12.3%	25.3%
	41-50	Count	45	37	82
		% within Age	54.9%	45.1%	100.0%
		% of Total	11.3%	9.3%	20.6%
	more than 51	Count	16	16	32
		% within Age	50.0%	50.0%	100.0%
		% of Total	4.0%	4.0%	8.0%
Total		Count	216	183	399
		% within Age	54.1%	45.9%	100.0%
		% of Total	54.1%	45.9%	100.0%

## Discussion

Based on the fact that cosmetic surgery media coverage is becoming more common lately, with higher numbers of adolescents and adults being affected and pursuing plastic surgery, the American and British Associations for Plastic Surgery have expressed solid concerns about the nature of this coverage [18-19]. Therefore, we conducted this current investigation to determine the role of social media in the decision-making process for cosmetic surgery. We investigated the role of four certain factors on the decision to undergo plastic surgery, which included the surgeon’s self-advertisement, cosmetic TV programs, preoperative and postoperative images on social media, and the need to look better in selfies.

More than half of our study population reported that the surgeon’s self-advertisement was a significant determinant of their decision to undergo cosmetic surgery, with the age group of 21-30 years being more affected than others. Moreover, females were more likely to be affected by the surgeon’s self-advertisement than males (63.2% vs. 44.1%), respectively. These findings go in line with a previous study that showed that nearly 60% of their population were affected by the surgeon’s posts on social media [20]. In another research, it was found that around 70% of people relied upon the quality of the surgeon’s website as the "most powerful" influencer on their choice of the plastic surgeon [21]. In general, social media may raise a patient’s sense of knowledge or confidence about a specific surgery (via online images, infographics on the surgeon’s website, other’s experiences/testimonials, and so on), without actually signifying their understanding of how the procedure might work for them or what the risks are [22]. That being said, when the outcomes of the surgery do not live up to the expectations of the patient, he/she feels betrayed not just by the plastic surgeon but also by the "brand" that the surgeon has built and eventually resulting in a phenomenon known as "institutional betrayal" [23-24]. Therefore, it’s advised that plastic surgeons should consider the likely impact of social media practices on the quality of life of those undergoing cosmetic surgery through an ethical lens.

Our analysis revealed that nearly half of the respondents reported being influenced by common cosmetic TV programs, with the age group of 31-40 years being the most affected. On the other hand, a study conducted among adolescent girls (14-18 years) found that single exposure to cosmetic TV shows resulted in dissatisfaction about the way they look, however, the statistical power was not high enough to significantly impact their attitude toward undergoing cosmetic surgery [25]. Meanwhile, our findings go in line with another study where exposure to cosmetic reality TV resulted in an increase in the desire to undergo surgery [26]. The reported attitudes toward cosmetic surgery in our study population are the outcomes of repeated exposure to cosmetic TV shows and not based upon a single exposure. Repeated exposure to TV shows could have led our respondents to believe that what they are watching is representative of reality [27].

The use of images of women who underwent cosmetic surgery is a commonly used method to attract those thinking about having plastic surgeries; it is widely used in media posts on cosmetic surgery (68%) [28]. In our investigation, a total of 65.7% of our respondents reported that their decision to undergo surgery was affected by pre- and postoperative images on social media, where females were more likely to be affected than males (69.7% vs 42.4%). Consistently, Walker et al. [29] reported that his population’s desire to undergo cosmetic surgery was largely affected by the images of females who underwent cosmetic enhancements and posted their images on social media. This also goes in line with a study performed on 100 patients in the US, which showed that 50% of the patients preferred seeing images before and after surgery, rather than watching illustrative videos, testimonials, or practice information [20].

More than half of our study population (54.1%) reported that the need to "look better in selfies and photos" was the main reason to undergo cosmetic surgery. This is also compatible with the findings of a recent study, which stated that 55% of plastic surgeons reported that wanting to look better in selfies was the main determinant of the decision of undergoing plastic surgery in the majority of the studies' population [30].

Furthermore, we noted that the group of participants between 21 and 30 years of age was the most vulnerable group to be influenced by all of the factors incorporated in our analysis except for cosmetic TV programs. This could be related to the higher frequency of watching TV programs, checking the images of those who underwent cosmetic surgery, and exploring the surgeon’s website, significantly impacting their decision to undergo further cosmetic surgery.

The present study adds significant value to the current literature with the larger sample size in our study compared to the previously published research on this topic. Also, we have noted an age-related pattern regarding the intention to undergo plastic surgery, which was not investigated by any prior research. That being said, more research is warranted in order to identify all of the factors related to the decision-making for cosmetic surgery as well as to determine any demographic-related patterns.

## Conclusions

The results of this study have shown that the majority of patients visiting plastic surgery clinics were positively affected, but not exclusively, by the media coverage of cosmetic surgery results. Consequently, social media, TV programs, and advertisements are capable of encouraging patients to undergo plastic surgeries, setting new trends in the world of cosmetic surgery, and spreading hope for severely deformed patients to restore the shape and function of their body parts. That being the case, the message through the media must always be realistic and focus on boosting the self-esteem of the community, embrace the different characteristics of the society, and reject the concept of ideal beauty. Moreover, the exposure of the youth to those TV shows and social media messages should be monitored to avoid their unwanted influence and unnecessary procedures.
